# Mood Responses Associated With COVID-19 Restrictions

**DOI:** 10.3389/fpsyg.2020.589598

**Published:** 2020-11-04

**Authors:** Peter C. Terry, Renée L. Parsons-Smith, Victoria R. Terry

**Affiliations:** ^1^Centre for Health Research, University of Southern Queensland, Toowoomba, QLD, Australia; ^2^School of Psychology and Counselling, University of Southern Queensland, Toowoomba, QLD, Australia; ^3^School of Social Sciences, University of the Sunshine Coast, Sippy Downs, QLD, Australia; ^4^School of Nursing and Midwifery, University of Southern Queensland, Toowoomba, QLD, Australia

**Keywords:** affect, emotion, COVID-19, pandemic, mood profiling, BRUMS

## Abstract

The COVID-19 pandemic resulted in more than half the world’s population being placed in lockdown to stem the spread of the virus. The severe restrictions imposed in many nations had the potential to significantly influence the physical and psychological well-being of those affected. The aim of the current study was to investigate mood responses during the period of restrictions from March to June, 2020. Mood responses of 1,062 participants (386 male, 676 female) were collected using the Brunel Mood Scale, hosted on the *In The Mood* website www.moodprofiling.com. The mean pattern of mood responses reflected an inverse iceberg profile, characterized by significantly elevated scores for tension, depression, anger, fatigue, and confusion, and below average scores for vigor; a profile associated with increased risk of mental health issues. Females reported more negative mood scores than males. Participants in the ≤25 age group reported the most negative profiles whereas those in the ≥56 age group reported the least negative profiles. Mood differences related to education status were also evident. Finally, mood scores fluctuated over time, with profiles being most negative during April and June. Overall, results confirmed significant mood disturbance during the period of COVID-19 restrictions, representing increased risk of psychopathology.

## Introduction

On the 30th January 2020, the World Health Organisation (WHO) declared the novel coronavirus 2019-nCoV (COVID-19) to be a “public health emergency of international concern” (World Health Organization, 2020a, p. 1)^1^. By the end of September 2020, COVID-19 had been contracted by over 35 million people globally and had caused more than 1 million deaths (Centre for Systems Science and Engineering, 2020)^2^. To interrupt the flow of transmission, significant restrictions were introduced, impinging on a large proportion of the world’s population. International traffic was affected, with many countries closing national borders and introducing overseas travel bans. Citizens were required to reduce daily contact and remain indoors for extended periods, colloquially referred to as “lockdown” (Hale et al., 2020), many small businesses were forced to close, financial markets retreated, and unemployment soared (Pak et al., 2020).

Given the unprecedented consequences of this global health crisis, investigating the effects of such wide-ranging restrictions on indicators of mental health is critically important. COVID-19 and other strains of coronavirus have been shown to inflict adverse mental health effects, not only on those who contract the disease (Rogers et al., 2020), but also on those placed in precautionary quarantine (Brooks et al., 2020), on health caregivers (Pappa et al., 2020), and on individuals whose daily lives are severely impacted (Ammar et al., 2020c).

A meta-analysis of 65 independent studies (Rogers et al., 2020) showed that individuals who had contracted but recovered from a severe coronavirus infection, including Severe Acute Respiratory Syndrome (SARS) and Middle East Respiratory Syndrome (MERS), were susceptible to mental health issues in the longer-term, including depression, anxiety, fatigue, and post-traumatic stress disorder (PTSD), sometimes years after being discharged from hospital. Moreover, an investigation of psychiatric complications among COVID-19 patients confirmed that the effects of the disease extend beyond respiratory issues in many cases, prompting a range of adverse cerebral events that include psychosis and affective disorders (Varatharaj et al., 2020).

A review of 24 studies investigating the psychological impact of being in quarantine (Brooks et al., 2020) also identified several negative effects, including PTSD symptoms, confusion, and anger. Fear, frustration, and boredom were among the stressors listed as contributing to mental health issues. Several predictions of a looming mental health crisis associated with COVID-19 have been promulgated (e.g., Pfefferbaum and North, 2020), along with a range of publications outlining the likely psychosocial effects of the pandemic with accompanying advice on how to manage mental health (e.g., World Health Organization, 2020b)^3^. A large-scale investigation of the psychosocial impacts of home confinement, involving 35 research organizations globally, identified significantly decreased life satisfaction associated with dramatic reductions in social participation through family, friends, and entertainment (Ammar et al., 2020b).

A systematic review and meta-analysis of 13 studies conducted since the COVID-19 pandemic commenced, covering a combined total of 33,062 healthcare workers (Pappa et al., 2020), found the prevalence of mental health issues, particularly depression and anxiety, to be significantly elevated compared to population norms, especially among females. Further, a multicenter study of the emotional consequences of COVID-19 lockdown, involving 35 research organizations globally, reported reduced overall mental well-being and increased depressive symptoms triggered by enforced home confinement (Ammar et al., 2020c). Moreover, a national survey of 13,829 respondents in Australia during the first month of COVID-19 restrictions (Fisher et al., 2020) concluded that mental health problems were at least twice as prevalent as in non-pandemic circumstances.

The effects of COVID-19 on the mood responses of individuals is an important indicator of how well society is coping with the pandemic. The YouGov website in the United Kingdom provides a weekly assessment of the mood of the nation, which showed that the percentage of those reporting feeling “happy” had plummeted from 50% in early March 2020 to 26% a month later, whereas those feeling “scared” had risen from a norm of 11 to 34%, feeling “bored” from 19 to 34%, and feeling “stressed” from 41 to 48% (YouGov, 2020)^4^. These data offer clear signs that the collective mood of the country deteriorated once lockdown measures were introduced into the United Kingdom.

Using a similar research paradigm to the YouGov approach, our study focused on assessing the mood responses of individuals during the period when movement and gathering restrictions were in place, and comparing the observed mood scores with well-established normative values developed prior to the COVID-19 outbreak (Terry et al., 1999, 2003a; Terry and Lane, 2010). For the purpose of our investigation, mood is defined as “a set of feelings, ephemeral in nature, varying in intensity and duration, and usually involving more than one emotion” (Lane and Terry, 2000, p. 17).

Several distinct mood profiles have been identified, based on the Profile of Mood States (McNair et al., 1971) or derivative measures, such as the Brunel Mood Scale (Terry et al., 1999, 2003a). For example, Morgan (1985) proposed that the *iceberg* profile, a pattern of mood responses characterized by above average scores for vigor and below average scores for tension, depression, anger, fatigue, and confusion, was associated with psychological well-being, whereas negative moods are associated with increased risk of psychopathology. Subsequently, Morgan et al. (1987) and others have highlighted the *inverse iceberg* mood profile, characterized by above average scores for tension, depression, anger, fatigue, and confusion, and below average scores for vigor, as indicative of increased risk of a range of pathologies, including chronic fatigue, overtraining syndrome, PTSD, and eating disorders (e.g., Budgett, 1998; Terry and Galambos, 2004; van Wijk et al., 2013).

More recent studies (Parsons-Smith et al., 2017; Quartiroli et al., 2018; Han et al., 2020) have identified new profiles, referred to as the *inverse Everest*, *shark fin*, *submerged*, and *surface* profiles. The inverse Everest profile is characterized by low vigor scores, high scores for tension and fatigue, and very high scores for depression, anger, and confusion. The shark fin profile is characterized by below average scores for tension, depression, anger, vigor, and confusion, combined with a high score for fatigue. The submerged profile is characterized by below average scores for all six mood dimensions. The surface profile is characterized by average scores for all six mood dimensions. In the present study, it was hypothesized that during the period of COVID-19-related restrictions there would be increased prevalence of inverse iceberg and inverse Everest profiles and decreased prevalence of iceberg and submerged profiles.

## Materials and Methods

### Participants

A total of 1,062 individuals participated in an online study. A range of age bands, ethnicities, and education levels were represented (see Table 1). Age bands were represented relatively evenly, but sex (64% female), ethnicity (80% Caucasian), and education level (59% university educated) were unevenly distributed.

**TABLE 1:** Sample demographics (*N* = 1,062).

Source	*n*	%
**Sex**		
Male	386	36.3
Female	676	63.7
**Age band (years)**		
≤25	243	22.9
26–35	263	24.8
36–45	232	21.8
46–55	167	15.7
≥56	157	14.8
**Ethnicity**		
African	16	1.5
Asian	87	8.2
Caucasian	853	80.3
Indigenous	18	1.7
Middle Eastern	19	1.8
Other	69	6.5
**Education level**		
≤High school graduate	238	22.4
TAFE^1^/Trade qualification	197	18.5
University qualification	316	29.8
Postgraduate qualification	311	29.3

*^1^TAFE, Technical and Further Education.*

### Measures

Participants reported relevant demographic information (sex, age band, ethnicity, education level) and completed the Brunel Mood Scale (BRUMS; Terry et al., 1999, 2003a). The BRUMS is a 24-item scale of basic mood descriptors, with a standard response timeframe of “How do you feel right now?” Participants rated their moods on a five-point Likert scale (0 = *not at all*, 1 = *a little*, 2 = *moderately*, 3 = *quite a bit*, and 4 = *extremely*). The BRUMS has six subscales (i.e., anger, confusion, depression, fatigue, tension, and vigor) each with four items. Total subscale scores range from 0 to 16. Raw scores are transformed into standard scores with reference to established tables of normative data (see Terry et al., 2003a). The BRUMS has been validated across diverse cultures (e.g., Terry et al., 2003b; Zhang et al., 2014; Han et al., 2020) and situational contexts (e.g., van Wijk et al., 2013; Sties et al., 2014). Good internal consistency has been demonstrated for the six subscales, with Cronbach alpha coefficients ranging from 0.74 to 0.90 (Terry et al., 1999).

### Procedure

All data were collected via the *In The Mood* website (Terry et al., 2013). The BRUMS takes approximately 2 min to complete. The website database has almost 28,000 completed BRUMS profiles. Data collected during the current study were compared with established norms. The study was conducted in accordance with the Australian Code for the Responsible Conduct of Research. The protocol was approved by the Human Research Ethics Committee at the University of Southern Queensland (approval number: H19REA100).

### Data Screening

As the website does not allow participants to submit the BRUMS for scoring unless all items have been answered, there were no missing values. Consistent with previous samples (e.g., Parsons-Smith et al., 2017; Quartiroli et al., 2018), univariate non-normality was evident for some subscales (e.g., depression, anger, and tension). As is typical of mood measures, negative scores tended toward higher numbers at the lower end of the scoring range, and lower numbers at the upper end (Terry et al., 1999, 2003a). Frequency distributions for skewness and kurtosis were examined and it was concluded that deviations from normal distribution were unlikely to make a substantive difference to the analyses, thus no data were removed. Using the Mahalanobis distance test (*p* < 0.001), a total of 13 multivariate outliers were identified, although a case-by-case inspection found no examples of response bias in the form of acquiescent, extreme, or straight line responding (Meisenberg and Williams, 2008; Leiner, 2019). Hence, all outliers were retained in the sample of 1,062 respondents.

## Results

### Mean Mood Profile During COVID-19 Restrictions

**FIGURE 1:**
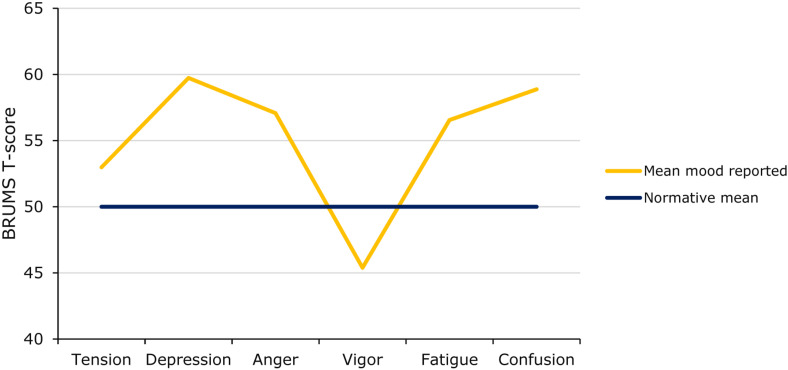
Mean mood profile reported during COVID-19 restrictions (*N* = 1,062).

The full range of raw scores (0–16) was observed for all six subscales. Once the raw scores were transformed into standard scores (T-scores), the mean mood profile of the whole sample, when plotted against relevant norms, represented an inverse iceberg profile (see Figure 1). The observed mean scores for all mood dimensions were significantly different from the normative mean score of 50 (*p* < 0.001; see Table 2). Effect sizes were small for tension scores (*d* = 0.28) and moderate-to-large for depression, anger, vigor, fatigue, and confusion scores (*d* = 0.54–0.70).

**TABLE 2:** Comparison of mean BRUMS scores vs. norms (*N* = 1,062).

Mood dimension	*M*	*SD*	Range	*t*	*d*
Tension	52.98	10.80	[40–83]	8.99^†^	0.28
Depression	59.74	14.83	[44–106]	21.40^†^	0.66
Anger	57.08	12.21	[45–98]	18.90^†^	0.58
Vigor	45.39	8.47	[31–71]	17.74^†^	0.54
Fatigue	56.56	10.55	[40–79]	20.26^†^	0.62
Confusion	58.88	12.76	[43–99]	22.68^†^	0.70

*t, t-test for difference between observed mean and normative mean of 50; d, effect size; ^†^p < 0.001.*

### Cluster Analysis

**FIGURE 2:**
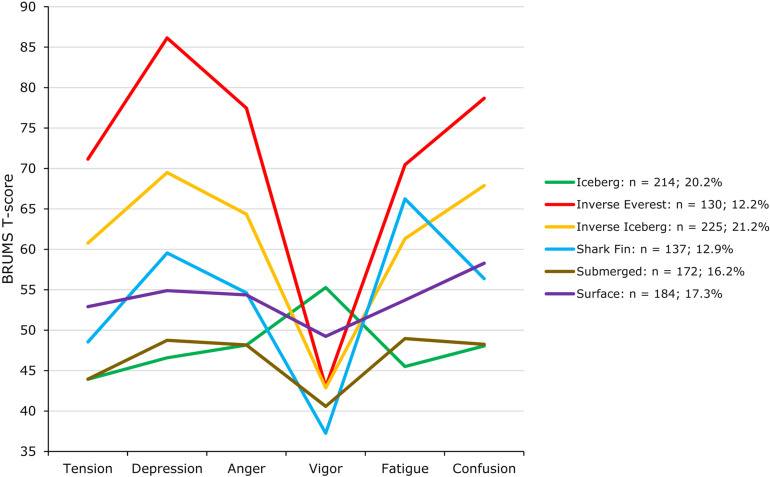
Prevalence of mood profile clusters (*N* = 1,062).

A seeded k-means cluster analysis with a prescribed six-cluster solution clearly identified the same six mood profiles previously reported in the literature (e.g., Parsons-Smith et al., 2017; see Figure 2). However, although the profiles were identical to those found in previous investigations, their prevalence was markedly different. Prevalence of the shark fin profile (12.9%) and surface profile (17.3%) was consistent with previous studies (∼15.1 [range = 13.0–17.3%] and ∼17.0 [range = 14.8–21%], respectively). However, as hypothesized, there were significantly fewer iceberg profiles (20.2 vs. ∼27.6% [range = 23.3–30.0%]) and submerged profiles reported (16.2 vs. ∼24.8% [range = 18.0–31.4%]). Most notably, and again as hypothesized, the inverse iceberg was the most commonly reported profile in the present investigation (21.2 vs. ∼11.9% [range = 9.3–14.0%]) and the inverse Everest profile was reported by 12.2% of participants compared to the typical ∼3.8% (range = 2.4–5.0%; Han et al., 2020; Parsons-Smith et al., 2017; Quartiroli et al., 2018; Terry and Parsons-Smith, 2019). Both the inverse iceberg and inverse Everest profiles reflect increased risk of psychopathology (e.g., Terry and Galambos, 2004; van Wijk et al., 2013).

### Demographic Influences on Mood Responses

Single-factor MANOVAs were used to investigate the influence of sex, age band, level of education, and month on mood responses and univariate analyses were used to identify significant between-group differences. Ethnicity was excluded from analyses due to unequal sample sizes (Tabachnick and Fidell, 2019). Significant multivariate variability at *p* < 0.001 was found for each variable analyzed (see Table 3).

**TABLE 3:** MANOVA of BRUMS subscales by demographic variables.

	Tension		Depression		Anger		Vigor		Fatigue		Confusion	
Source	*M*	*SD*	*M*	*SD*	*M*	*SD*	*M*	*SD*	*M*	*SD*	*M*	*SD*
**Sex [*T^2^* = 0.13, *F*(6,1055) = 23.04^†^]**												
Male (*n* = 386)	51.27^†^	10.08	58.02*	14.13	57.91	12.72	48.46^†^	8.09	54.05^†^	9.05	57.23^†^	12.24
Female (*n* = 676)	53.96	11.08	60.72	15.13	56.61	11.89	43.63	8.19	58.00	10.85	59.82	12.97
**Age band [*T*^2^ = 0.08, *F*(24,4202) = 3.60^†^]**												
≤25 years^a^ (*n* = 243)	55.24^†e^	11.87	62.03^†e^	15.50	57.80*^e^	12.05	45.65	7.99	58.63^†e^	10.39	62.27^†de^	13.36
26–35 years^b^ (*n* = 263)	54.04^†e^	10.74	60.94*^e^	15.34	57.96*^e^	13.06	45.08	8.94	57.27^†e^	10.60	59.90^†e^	12.84
36–45 years^c^ (*n* = 232)	53.06	10.15	60.09	14.15	58.42^†e^	12.07	44.48	8.54	57.68^†e^	10.66	58.80	12.08
46–55 years^d^ (*n* = 167)	51.25*^a^	10.51	58.05	14.94	56.19	13.28	45.24	8.39	55.22	10.34	56.50	12.57
≥56 years^e^ (*n* = 157)	49.44	9.20	55.46	12.69	53.46	8.94	46.99	8.23	51.95	9.25	54.57	11.21
**Education level [*T^2^* = 0.05, *F*(18,3155) = 2.92^†^]**												
≤High school^a^ (*n* = 238)	52.86	10.94	60.72	14.96	57.48	12.28	44.69	8.29	57.06	10.63	58.96	12.33
TAFE^1^/Trade^b^ (*n* = 197)	53.88	11.59	61.82*^d^	15.41	58.06	12.36	43.24*^c^	7.86	59.49^†d^	11.45	60.50	12.99
University^c^ (*n* = 316)	53.36	10.85	60.07	15.10	57.06	12.58	45.80	8.28	56.61	10.45	59.67	13.58
Postgraduate^d^ (*n* = 311)	52.12	10.07	57.33	13.79	56.17	11.66	46.86^†b^	8.88	54.28	9.46	56.99	11.89
**Month [*T^2^* = 0.06, *F*(18,3155) = 3.65^†^]**												
March 2020^a^ (*n* = 33)	51.45	8.34	50.67^†bd^	10.13	51.36	8.03	48.24	7.55	49.61^†bd^	8.20	54.82	9.29
April 2020^b^ (*n* = 633)	53.23	10.99	60.50	15.12	57.14	12.05	44.66^†c^	8.19	57.26*^c^	10.69	59.23	13.07
May 2020^c^ (*n* = 185)	51.63	10.14	56.82	13.67	54.83	11.07	47.54	9.45	54.42	9.79	56.52	11.95
June 2020^d^ (*n* = 211)	53.65	11.06	61.44	14.77	59.76^†ac^	13.48	45.22	8.20	57.45	10.48	60.52	12.65

T^2^, Hotelling’s T-squared; ^†^p < 0.001; *p < 0.008; ^1^TAFE, Technical and Further Education. Superscript letters a–e are used to indicate sub-group differences.

Univariate differences were assessed using a Bonferroni-adjusted alpha level of *p* < 0.008. Females reported higher scores for tension, depression, fatigue, and confusion, and lower scores for vigor, compared with males. Those aged ≤25 years reported higher scores for tension and confusion compared with those aged from 46 to 55 years. Participants in the ≥56 category scored lower for tension, depression, anger, fatigue, and confusion compared with the ≤25 and 26–35 age bands, as well as lower scores for anger and fatigue compared with individuals aged 36–45 years. For education, participants with a TAFE/trade qualification scored higher for depression and fatigue compared with those with a postgraduate qualification, and lower for vigor in comparison to individuals with either a university or postgraduate level of education. In terms of trends over time, participants scored lower for depression and fatigue in March compared with April and June. Lower vigor and higher fatigue scores were reported in April compared with May. Higher anger scores were reported in June compared with March and May.

### Distribution of Mood Profiles by Demographic Variable

Chi-squared tests were used to assess the distribution of mood profile clusters by demographic variables of interest. Significant associations between the six mood profiles and sex, age group, and education level were found (see Table 4). Adjusted residuals were assessed against the critical values of ±1.96, ±2.58, and ±3.29 (Field, 2009) to identify the source of differences.

**TABLE 4:** Distribution of clusters by demographic variables.

	Cluster											
Source	1	%	2	%	3	%	4	%	5	%	6	%
**Sex [χ^2^(5,1062) = 52.07^†^]**												
Male (*n* = 386)	111^†+^	28.8	38	9.8	71	18.4	27^†–^	7.0	55	14.2	84^§+^	21.8
Female (*n* = 676)	103^†–^	15.2	92	13.6	154	22.8	110^†+^	16.3	117	17.3	100^§–^	14.8
**Age band [χ^2^(20,1062) = 80.13^†^]**												
≤25 years (*n* = 243)	31^†–^	12.8	39*^+^	16.0	52	21.4	36	14.8	27*^–^	11.1	58^§^^+^	23.9
26-35 years (*n* = 263)	41*^–^	15.6	37	14.1	58	22.1	35	13.3	43	16.3	49	18.6
36-45 years (*n* = 232)	39	16.8	30	12.9	61*^+^	26.3	28	12.1	40	17.2	34	14.7
46-55 years (*n* = 167)	41	24.6	15	9.0	31	18.6	21	12.6	39^§+^	23.4	20	12.0
≥56 years (*n* = 157)	62^†+^	39.5	9^§–^	5.7	23*^–^	14.6	17	10.8	23	14.6	23	14.6
Education level [χ^2^(15,1062) = 28.99*]												
≤High school (*n* = 238)	43	18.1	32	13.4	51	21.4	36	15.1	41	17.2	35	14.7
TAFE^1^/Trade (*n* = 197)	29*^–^	14.7	34*^+^	17.3	41	20.8	32	16.2	31	15.7	30	15.2
University (*n* = 316)	56	17.7	36	11.4	73	23.1	35	11.1	52	16.5	64	20.3
Postgraduate (*n* = 311)	86^†+^	27.7	28*^–^	9.0	60	19.3	34	10.9	48	15.4	55	17.7

1, Iceberg; 2, Inverse Everest; 3, Inverse Iceberg; 4, Shark Fin; 5, Submerged; 6, Surface; ^+^, over-represented, ^–^, under-represented; ^†^p < 0.001; ^§^p < 0.01; *p < 0.05; ^1^TAFE, Technical and Further Education.

#### Sex

The distribution of mood profiles varied significantly by sex, with males generally reporting more positive profiles. Males were over-represented in the iceberg profile whereas females were over-represented in the shark fin profile, consistent with previous studies (Parsons-Smith et al., 2017; Quartiroli et al., 2018; Han et al., 2020). Males were over-represented in the surface profile compared with females, consistent with Han et al. (2020). Although females reported a higher prevalence of inverse iceberg profiles, the distribution did not vary significantly, mirroring the findings of Quartiroli et al. (2018). The distributions of the inverse Everest and submerged profiles were independent of sex.

#### Age Band

A general trend of mood profiles being more positive among older age groups was evident, largely consistent with previous age group comparisons (Parsons-Smith et al., 2017; Quartiroli et al., 2018). Younger participants (≤25 years, 26–35 years) were under-represented and older participants (≥56 years) over-represented in the iceberg profile. Younger participants (≤25 years) were over-represented and older participants (≥56 years) under-represented in the inverse Everest profile. Participants aged 36–45 years were over-represented in the inverse iceberg profile, whereas those ≥56 years were under-represented. Individuals aged 46–55 years were over-represented in the submerged profile, whereas those ≤25 years were under-represented. The distribution for the shark fin profile was independent of age.

#### Level of Education

Participants with a TAFE/trade qualification were under-represented in the iceberg profile and over-represented in the inverse Everest profile. The reverse was true for those with a postgraduate level of education. Distributions for the inverse iceberg, shark fin, submerged, and surface profiles were independent of level of education.

## Discussion

The mean mood profile for the participant group collectively, compared to normative scores, was characterized by elevated tension, depression, anger, fatigue, and confusion, and reduced vigor. Significant mood disturbance was further reflected in the prevalence of mood profile clusters, when compared to prevalence rates reported in previous studies. For example, the inverse iceberg was reported by 21.2% of participants and the inverse Everest profile by 12.2% of participants, compared to the typical prevalence of 11.9% and 3.8%, respectively (Parsons-Smith et al., 2017; Quartiroli et al., 2018; Terry and Parsons-Smith, 2019; Han et al., 2020). This suggests that ∼33% of our sample were at increased risk of experiencing some form of clinically diagnosable mood-related disorder, whereas the global point prevalence of mood disorders based on the results of 148 studies is 5.4% (Steel et al., 2014). Our findings align with those of Fisher et al. (2020) who found that 25% of participants reported mild to moderate depressive symptomology during the first month of COVID-19 restrictions.

There are several plausible explanations for the observed increase in negative feeling states. The pandemic has undoubtedly caused fear and loss for many individuals; health fears for self and loved ones, fear of isolation, loss of income, social support, and a sense of normality, the list is extensive. The notion of disenfranchised grief (Doka, 2002) offers a potential explanation for the widespread mood disturbance evident among participants. Grief at the loss of someone or something dear to an individual is said to be disenfranchised when the grief is perceived to be unacknowledged or unworthy. During the pandemic, many individuals have lost livelihoods, relationships and opportunities, or been denied access to simple things that give them pleasure, such as physical contact with friends and family, a trip to the local café, or interacting with work colleagues. Although such losses can trigger a genuine grief response, knowledge of countless pandemic-related deaths may create a perceived obligation to minimize the outward expression of loss because others are in far worse circumstances. A reluctance or inability to share grief and loss with others may be associated with mood decrements and increased potential for psychopathology (Fisher et al., 2020).

Mood disturbance may also be explained by reduced physical activity and increased sedentary behaviors during COVID-19 restrictions. The antidepressant effect of exercise has a strong evidence base (Dunn et al., 2005; Siqueira et al., 2016) and exercise as a treatment for mood disorders is also well established (Hearing et al., 2016). The National Physical Activity Guidelines for Adults advocates a simple message of *moving more and sitting less*, with a recommendation to accumulate 150–300 min/week of moderate intensity physical activity or 75–150 min/week of vigorous exercise (Department of Health, 2019)^5^. Unfortunately, since COVID-19 restrictions have come into force, many people have been *moving less and sitting more* (Ammar et al., 2020a). Moreover, reduced exercise duration during the pandemic has been associated with higher scores for depression, anxiety, and stress (Stanton et al., 2020). Encouragingly, some recently published papers have offered guidelines and practical recommendations for staying physically active during quarantine and/or self-isolation (e.g., Bentlage et al., 2020; Chtourou et al., 2020).

Trait characteristics may also play an important role in determining mood responses to COVID-19 restrictions. An Italian study conducted during the early stages of the pandemic in Europe (February–March, 2020) among a sample of 2,886 participants (Pagnini et al., 2020) showed that negative feeling states in response to movement restrictions were more common among those with greater cognitive rigidity and emotional instability.

Results of between-group comparisons identified similar findings to those reported previously. Compared to males, females reported significantly higher levels of tension, depression, fatigue, and confusion, together with lower levels of vigor, replicating the findings of Han et al. (2020). Research on the six mood profile clusters has consistently found an increased prevalence of the more negative mood profiles for females compared with males (Parsons-Smith et al., 2017; Quartiroli et al., 2018; Han et al., 2020), and the Australian Bureau of Statistics (2008)^6^ notes that females are almost twice as likely as males to be affected by a mood disorder (8.4 vs. 4.3%).

Several explanations have been advanced to explain sex differences in mood responses. From a chronobiological perspective, there is evidence to support a sex-specific predisposition to depressive states. Many sub-threshold depressive symptoms, and indeed mood disorders, have been tentatively linked to dramatic hormonal fluctuations relating to reproductive-related events (e.g., menarche, menstruation, pregnancy, postpartum, menopause; Soares, 2013). Such “windows of vulnerability” (Soares, 2013, p. 677) are thought to predispose women to depressive symptoms via estrogen-serotonin interactions (Miller et al., 2002; Amin et al., 2005). Estrogen has been found to play an important mechanistic role in mood regulation (Halbreich and Kahn, 2001; Miller et al., 2002), although the specific pathophysiological pathways remain poorly understood (Soares, 2013). Other explanations are psychological in nature, including sex differences in ability to downregulate negative feeling states through the implementation of effective strategies (Nolen-Hoeksema, 1991, 2012), and a greater willingness among females to report mood disturbance (Bogner and Gallo, 2004).

Regarding age, it is evident globally that those in the 18–25 age group have been disproportionately affected materially by the pandemic, in terms of reduced employment and income (Belot et al., 2020). Logically, such detrimental effects would act as a catalyst for mood disturbance among younger individuals. However, nuanced differences in the adoption of effective emotion-regulation strategies may also underlie age-related variations in reported mood. Consistent with previous findings (Parsons-Smith et al., 2017; Quartiroli et al., 2018; Han et al., 2020), younger participants reported higher scores for tension, depression, anger, fatigue, and confusion compared with their older counterparts, and were more likely to report negative mood profiles, rather than the iceberg profile more frequently reported by those aged ≥56 years. Associations between maladaptive coping strategies and psychopathology symptom development have been reported (McLaughlin et al., 2011). Younger adults are more likely to utilize rumination, avoidance, and suppression, all of which are associated with poorer mental health outcomes (Aldao et al., 2010). Further, a reciprocal relationship exists between rumination and development of depression and anxiety symptomology (McLaughlin and Nolen-Hoeksema, 2011).

Given the saturation of negative COVID-19 information in the media, younger adults may find it difficult to employ cognitive distraction and avoidance strategies and more likely to engage in maladaptive emotion-regulation strategies, such as rumination and suppression of feelings. Conversely, adaptive strategies such as acceptance, reappraisal, and problem solving, which are associated with more positive outcomes, are techniques more often adopted by older adults (Aldao et al., 2010). Additionally, older adults are more likely to have built a repertoire of effective and flexible coping strategies from which to draw that may better suit challenging situations (Livingstone et al., 2020). Older adults may therefore be inclined to put COVID-19 restrictions into a broader and more manageable perspective. In general, active as opposed to passive emotion-focused strategies tend to be more adaptive and likely to be associated with reduced mood disturbance in the current climate.

In terms of level of education, participants with a postgraduate qualification reported lower scores for depression and fatigue and higher scores for vigor compared to those with a TAFE/trade qualification. These mean differences also translated into the postgraduate group being over-represented for the iceberg profile and under-represented for the inverse Everest profile, with the reverse being true for the TAFE/trade group. A clear link between education, income, and financial stress has been identified in the literature. In Australia, individuals with a doctoral degree are up to six times more likely to be in the top 10% of income earners, even after controlling for age, occupation, labor force status, and gender. Further, those with higher levels of education are more likely to be employed, and less likely to experience financial stress (Department of Education, Skills and Employment, 2020)^7^.

Variations in mood scores were also evident over time. Participants scored lower for depression and fatigue in March compared with April and June. Lower vigor and higher fatigue scores were also reported in April compared with May. A study from India conducted during the early stages of the COVID-19 pandemic provided insights into the mood of the population derived from the emotional content of more than 86,000 Twitter posts (Venigalla et al., 2020). The emotional content of tweets varied according to specific trigger events, such as the introduction and extension of lockdown restrictions. The mood fluctuations over time evident in our study appear to similarly reflect an emotional rollercoaster among participants, triggered by events such as the varying geographical spread and control of the virus, the dramatic economic fallouts, and the differential tightening and easing of restrictions.

Some limitations of our study are acknowledged. Online surveys require access to a computer with internet access and, in our case, fluency in English, which tends to reduce participation by those from lower socio-economic and marginalized groups, and non-English speakers. Further, the demographic characteristics of our sample showed an over-representation of females, Caucasians, and university-educated participants, which may limit the generalizability of the findings. It should also be noted that the BRUMS, as a brief measure of current mood, is not a diagnostic tool and hence, although our results may signal an increased risk of clinical psychopathology among participants, they could equally be seen in terms of challenging but essentially normal psychological adjustments to, in most people’s experience, unprecedented societal restrictions.

In summary, evidence regarding the economic impact of COVID-19, suggests that females, younger people, and lesser educated, lower paid individuals are at “the epicenter of the crisis” (Gustafsson and McCurdy, 2020, p. 9). Our findings indicate that these same groups are also experiencing the greatest emotional burden, in terms of mood disturbance.

## Conclusion

Clear evidence of elevated tension, depression, anger, fatigue, and confusion, and reduced vigor were identified, representing significant mood disturbance, and increasing the prospect of a forthcoming mental health crisis. An important implication of our findings is that urgent measures should be considered to ameliorate the negative impact of the COVID-19 pandemic on mental health.

## Data Availability Statement

The raw data supporting the conclusions of this article will be made available by the authors, without undue reservation.

## Ethics Statement

The studies involving human participants were reviewed and approved by the Human Research Ethics Committee, University of Southern Queensland, Australia (approval number: H19REA100). The patients/participants provided their written informed consent to participate in this study.

## Author Contributions

All authors listed have made a substantial, direct, and intellectual contribution to the work, and approved it for publication.

## Conflict of Interest

The authors declare that the research was conducted in the absence of any commercial or financial relationships that could be construed as a potential conflict of interest.

## Footnotes

1 https://www.who.int/news-room/articles-detail/updated-who-recommendations-for-international-traffic-in-relation-to-covid-19-outbreak

2 https://coronavirus.jhu.edu/map.html

3 https://www.who.int/docs/default-source/coronaviruse/mental-health-considerations.pdf?sfvrsn=6d3578af_10

4 https://yougov.co.uk/topics/science/trackers/britains-mood-measured-weekly

5 https://www1.health.gov.au/internet/main/publishing.nsf/Content/health-pubhlth-strateg-phys-act-guidelines

6 https://www.abs.gov.au/AUSSTATS/abs@.nsf/Lookup/4326.0Main+Features32007?OpenDocument

7 https://docs.education.gov.au/collections/benefits-educational-attainment
